# The Rabietis Bacillus

**Published:** 1899-04

**Authors:** 


THE RABIETIS BACILLUS. Foll, Rivolta, and Spinelli claimed to have detected a bacillus, the specific character of which was made highly probable by Sanfelice, who, employing a special staining process, demonstrated its presence in large numbers in the spinal cord of a boy dead from hydrophobia. Memmo, of Rome, confirmed the observation of Sanfelice, and proved the virulent character of the micro-organism, which he detected in several cases of rabies; but quite recently he has succeeded in cultivating it in artificial media, and in reproducing by inoculations with these cultures the disease, with its distinctive characters, in dogs, rodents, and birds, respectively. These experiments and observations which, fulfilling the most stringent demands of bacteriogical criticism, leave no room for doubt, are described in the *Centralblatt f. Bakteriologie.* He found the microbe in the cerebro-spinal fluid and the substance of the brain and spinal cord, in the saliva and parotid gland, and in the aqueous humor of four dogs dying from the natural disease, and of rabbits, guinea-pigs, and pigeons in which it had been produced by inoculation. It grew better in fluid than in solid media, the best being bouillon with glucose slightly acidulated with tartaric acid. The growth did not become manifest under a week, and was easily arrested by air-infection, as through the access of dust from the laboratory. Inoculated with cultures of the fourth generation, purified by successive plate-cultures, guinea-pigs and rabbits developed symptoms of paralysis of the hind limbs, etc., after from eleven to twenty days, and died from the paralytic form of the disease. With dogs the inoculation was prolonged to from thirty to sixty days, when they were attacked with all the symptoms of typical natural rabies. Though Memmo does not suggest the application of his discovery to the prophylaxis of hydrophobia, it is not impossible that artificial cultures may be found capable of definite attenuation so as ultimately to simplify the methods adopted by Pasteur.—London *Lancet*, September, 17 ; *Journal of the American Medical Association; The Pennsylvania Medical Journal*, Pittsburg, December, 1898.

